# Outbreak investigation: transmission of COVID-19 started from a spa facility in a local community in Korea

**DOI:** 10.4178/epih.e2020056

**Published:** 2020-07-29

**Authors:** Taewon Han

**Affiliations:** Local Epidemic Intelligence Officer and Public Health Doctor, Gyeongsangnam-do, Korea

**Keywords:** COVID-19, Transmission, Disease outbreak, Contact tracing, Korea

## Abstract

**OBJECTIVES:**

In Korea, there have been 10,480 confirmed cases of coronavirus disease 2019 (COVID-19) as of April 11, 2020. We investigated the transmission of COVID-19 in a cluster of cases.

**METHODS:**

We analyzed the epidemiological characteristics of 10 confirmed COVID-19 patients in an outbreak that started at Spa facility A in a local community in Korea on March 28, 2020 and traced them through April 8, 2020. Epidemiological surveys and diagnostic tests were conducted for each contact, and the secondary attack rate was estimated.

**RESULTS:**

There were 3 male confirmed patients (30.0%) and 7 female confirmed patients (70.0%), and their mean age was 53.5 years (range, 2.0 to 73.0). Two patients (20.0%) were asymptomatic. The incubation period was between 3 days and 12 days. Three confirmed patients were infected at female’s Spa facility A and 7 confirmed patients were second, third, and fourth generations of transmission. Seven confirmed patients contracted COVID-19 through presymptomatic contact. In total, 192 contacts were identified, with a secondary attack rate of 3.6%. Eighty-three contacts (43.2%) were aged 40-59 years, and the secondary attack rate was the highest (12.1%) in those aged ≥60 years. Most exposures (n=156, 81.3%) involved casual contact. The number of visitors using the female’s spa facility was 58, including 3 confirmed patients, resulting in a secondary outbreak rate of 5.9%.

**CONCLUSIONS:**

This study presents a cluster of cases occurring in a setting with high temperature and humidity. The second, third, and fourth generations were transmitted through presymptomatic contact.

## INTRODUCTION

The coronavirus disease 2019 (COVID-19) outbreak, which began in Wuhan, China, in December 2019, has spread throughout the world. On March 11, 2020, the World Health Organization declared COVID-19 a pandemic. COVID-19 causes symptoms such as fever, cough, and muscle pain, and the number of confirmed cases and deaths due to COVID-19 are rapidly increasing worldwide [1]. In Korea, there were 10,480 confirmed cases and 211 deaths due to COVID-19 as of April 11, 2020, after a person who entered the country from Wuhan on January 20, 2020 was confirmed with COVID-19. Most confirmed cases from the end of February until early April were reported in the Daegu and Gyeongsangbuk-do Provinces, which the government declared as special disaster zones. Today, sporadic cases of COVID-19 are still reported across the country [2].

Numerous studies conducted worldwide on COVID-19 have provided important findings regarding the natural history and transmission routes of COVID-19; however, there are still some questions to be answered. One of these is the possibility of asymptomatic or presymptomatic transmission and its influence [3]. COVID-19 has a higher early viral load than other known coronaviruses. Asymptomatic patients have a similar viral load as symptomatic patients, suggesting that the virus can be transmitted during the asymptomatic or early infection period [4]. If COVID-19 can be transmitted to humans before its symptoms manifest, it is necessary to promptly test and isolate the patients exhibiting symptoms, actively seek patients confirmed with COVID-19 who are in the presymptomatic period stage or show mild symptoms, and quarantine those who were in contact with confirmed cases. However, these measures are difficult to practically execute in the real world [3].

In this study, we investigated the degree to which the spread of COVID-19 can be controlled through the analysis of presymptomatic transmission patterns and contact tracing based on an outbreak that occurred in a local community in Region A, Korea. We also examined the characteristics of the COVID-19 outbreak that occurred in the same local community by examining those who were found to have been in contact with a confirmed case of COVID-19 through contact tracing and determining the COVID-19 secondary attack rate.

## MATERIALS AND METHODS

### Background and setting

A large number of confirmed cases of COVID-19 were reported in Region A between March 28, 2020 and March 31, 2020, and the outbreak was confirmed to be associated with Building A located in Region A and Spa facility A. Region A is a city with a population of approximately 350,000 and acts as the administration, traffic, and residence center for the surrounding cities. Aside from two confirmed cases from Sinchonji Church in Daegu that occurred 40 days prior to the outbreak associated with Spa facility A, no other confirmed case was reported in Region A. Building A has three stories below and 19 stories above the ground, with commercial facilities (first basement floor until the first floor) and knowledge industry centers (2nd to the 19th floor). There are approximately 2,500 residents in the building, and 2,300 cars enter and exit the building per day. The building has a floating population of approximately 350 people per day, including visitors of the commercial facilities. Spa facility A is located on the first basement floor of Building A. The facility has separate baths for female and male and has no mixed baths. Approximately 250 people visit the spa facility every day, and many are regular users of the spa facility and the accompanying gym. Many people from other regions visit the facility on the weekends. Public health centers and Provincial epidemiological investigation team formed a joint epidemiological investigation team to identify patient zero of the outbreak and contact trace those in contact with the confirmed cases.

### Epidemiologic investigation and response measures

Patient #1, who was the index case, visited Clinic A for symptoms including fever, muscle pain, and lethargy that started on March 27, 2020. Following the doctor’s advice that she needed to be tested for COVID-19, the patient visited a screening center at a public health center and was diagnosed with COVID-19 on March 28, 2020. The source of infection was unclear as the patient had not visited the special disaster zones (Daegu and Gyeongsangbuk-do Provinces) or any foreign country and had not encountered a confirmed case. On March 31, 2020, Patient #3, who had been tested at the screening center of a hospital for fever and lethargy that started five days ago, and Patient #4, the spouse of Patient #3 who had fever and muscle pain that started three days earlier were confirmed with COVID-19 at the same time. The source of infection was also unclear for Patients #3 and #4 since they had no epidemiological links to COVID-19, as was the case for Patient #1. Patients #5 to #10 were later confirmed with COVID-19 through contact tracing. After a comprehensive epidemiologic investigation identifying the patient zero of the outbreak, it was found that Patients #1, #3, and #4 regularly visited the spa facility inside Building A (Spa facility A), and that Patients #4 and #5’s workplaces were in Building A. The outbreak was thus deemed to be associated with Building A; accordingly, a screening test and epidemiologic investigation were conducted on the visitors of Building A. Patient #2 was confirmed with COVID-19 on April 3, 2020. Building A was closed on March 31, 2020 after the outbreak was reported, and a temporary screening center was created in front of the building to provide screening examinations and tests for the residents and visitors of the building between April 1, 2020 and April 5, 2020. Visitors from outside of Region A were informed to be tested for COVID-19 at a nearby public health center via disaster text alerts. An epidemiologic investigation was conducted on all the visitors of Building A from March 12, 2020, which was 14 days before the date of symptom onset for Patient #3 with the earliest symptom onset, and March 31, 2020, which was the date of the building closure. The following groups of people were tested: Group 1) people who were employees, visitors, or residents of Building A between March 12, 2020 and March 31, 2020 and showed symptoms of COVID-19; Group 2) visitors of Spa facility A between March 12, 2020 and March 24, 2020; Group 3) people who worked on the same floor as the office of the confirmed cases between March 27, 2020 and March 31, 2020; and Group 4) those in contact with Patients #1 to #10. COVID-19 was diagnosed based on the results of the real-time reverse transcription polymerase chain reaction (RT-PCR) assay. Samples were collected from the upper respiratory tract (nasopharynx, oropharynx) and lower respiratory tract (sputum). No sputum was collected from the lower respiratory tract if the patient had no sputum. Those confirmed with COVID-19 were transferred to a hospital specializing in COVID-19 and were subjected to an epidemiologic investigation. Of the people who tested negative for COVID-19, those who belonged to Group 1 were quarantined until their symptoms disappeared, those in Group 2 were quarantined until their test results were out, and those in Groups 3 and 4 were quarantined until 14 days after the last day of exposure. A comprehensive epidemiologic investigation was conducted using the global positioning system (GPS) data, card statements, and in-depth interviews of Patients #1, #2, and #3 to investigate the route of transmission of COVID-19. People who visited Spa facility A (for female) at around the same hours as Patients #1, #2, and #3 were identified using closed circuit television (CCTV) records and facility entry records and considered as visitors. An epidemiologic investigation was conducted among these people via phone calls in addition to tests for COVID-19.

### Case definition

A confirmed case was defined as a person who tested positive for COVID-19. A symptomatic contact was defined as someone who was in contact with a confirmed case showing symptoms. A presymptomatic contact was defined as someone who was in contact with a confirmed case who did not show symptoms at the time of the contact but showed symptoms thereafter. An asymptomatic patient was defined as a patient who showed no symptoms from the time of contact until a COVID-19 diagnostic test where the patient tested positive for COVID-19. An asymptomatic contact was defined as someone who was in contact with an asymptomatic patient. Symptomatic, presymptomatic, and asymptomatic transmission were defined as transmission of COVID-19 from a symptomatic, presymptomatic, and asymptomatic contact, respectively. Contact and transmission from a contact were determined based on the date of the first contact. Close contact: household family was defined as someone who was in contact with a family member with COVID-19 with whom the person lived. Close contact: travel was defined as someone who was in close contact with a confirmed case for over three hours as they traveled to another region aside from Region A. Close contact: meal was defined as someone who was in close contact with a confirmed case for over 30 minutes after having a meal together. A casual contact was defined as someone who spent several minutes with a confirmed case within the same space without any mask on (or a person was established as a contact by an Epidemic intelligence Officer). If such classifications overlapped, they were classified in the order of family, travel, meal, and casual contact. Contacts of COVID-19 patients were defined as those who were found to have been in contact with a confirmed case regardless of symptoms and were subjected to a diagnostic test for COVID-19. Those who tested negative were quarantined for 14 days from the last day of contact with a confirmed case. Additional tests were performed if new symptoms occurred, or symptoms changed during the quarantine period. Family members and healthcare workers were subjected to a diagnostic test for COVID-19 before a contact was discharged.

### Data collection and analysis

A standardized epidemiologic investigation format provided by the Korea Centers for Disease Control and Prevention and revised by a public healthcenter was used. Objective data were obtained from the GPS, card statements, and CCTV records. Information about epidemiologic links to COVID-19 and symptoms was obtained through phone interviews. The collected data were then analyzed.

### Ethics statement

The investigation was a part of public health response and was not considered research subject to institutional review board approval.

## RESULTS

**Figure 1.**
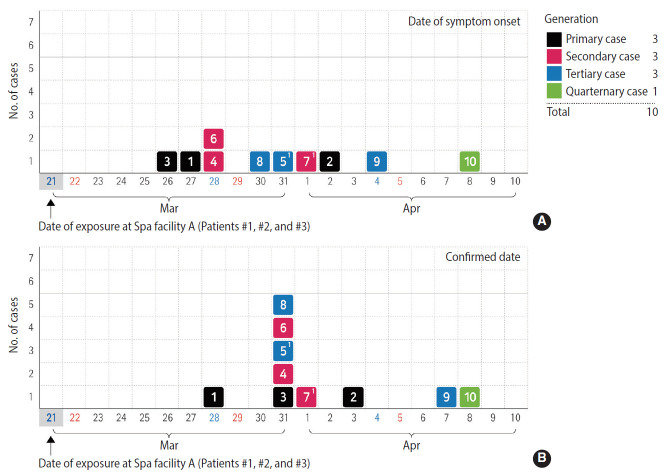
Epidemic curve of a coronavirus disease 2019 (COVID-19) outbreak in local community by date of symptom onset (A) and confirmed date (B) in 2020. ^1^For asymptomatic patients (Patients #5 and #7), the date of sample collection was set as the date of symptom onset.

From March 28, 2020 and April 3, 2020, a total of eight confirmed cases designated as Patients #1 to #8 occurred. Patients #9 and #10 who showed symptoms of COVID-19 and were being quarantined during this period were later tested and were confirmed as having COVID-19. Thus, a total of 10 confirmed cases were included in this study. Figure 1 shows an epidemic curve showing the outbreak progression. Of the 2,843 people who visited Building A between March 12, 2020 and March 31, 2020, 2,245 were tested for COVID-19. One person (Patient #2) was diagnosed with COVID-19 on April 3, 2020. The 192 people were found to have come in contact with the confirmed cases in an epidemiologic investigation, and seven of them were diagnosed with COVID-19. Table 1 shows the clinical and epidemiological characteristics of the 10 patients from the outbreak associated with the spa facility. Three patients were male (30.0%), and seven were female (70.0%). The mean age of the patients was 53.5 years (range, 2.0 to 73.0), and two (20.0%) were asymptomatic. The incubation period of COVID-9 was 3-12 days. The number of contacts ranged from 0 to 37. Of these, 0-14 people were classified as close contacts. Fever was the most common symptom, followed by cough, sore throat, and chills. Three confirmed cases (Patients #1, #2, and #3) were found to have visited Spa facility A (for female) inside Building A at around the same time in the epidemiologic investigation.

**Figure 2.**
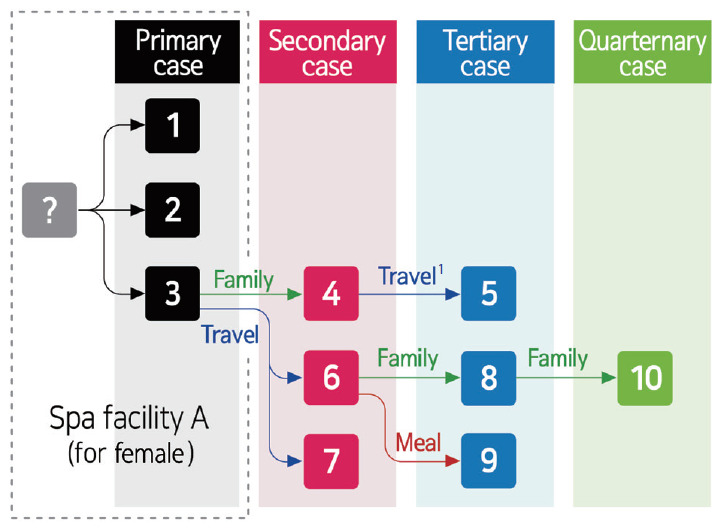
Contact history of the epidemiologically linked patients with coronavirus disease 2019 (COVID-19). ^1^Based on an epidemiologic investigation, Patients #4 and #5 were found to be from the same workplace. However, both patients only encountered each other on March 26, 2020 while they traveled together to another region.

Figure 2 shows the suspected routes of transmission for the 10 patients. It is suspected that Patients #1, #2, and #3 were exposed to an unidentified patient zero at Spa facility A (for female) and were infected with COVID-19 on March 21, 2020. Patient #3 transmitted the virus to Patient #4 (household family), Patient #6 (travel), and Patient #7 (travel). Patient #4 transmitted the virus to Patient #5 (travel). Patient #6 transmitted the virus to Patient #8 (household family) and Patient #9 (meal), and Patient #8 transmitted the virus to Patient #10 (household family). There was no secondary transmission by Patients #1 and #2.

Table 2 shows the characteristics of different contact types and the secondary attack rate among the 192 people who were in contact with a confirmed case of COVID-19. The mean secondary attack rate was 3.6%. Most of the contacts were females (32.9% male, and 67.2% female). The secondary attack rate was 4.8% for male and 3.1% for female. Most contacts (n = 83, 43.2%) were aged 40-59 years. However, the secondary attack rate was the highest at 12.1% for those aged 60 years or older. Casual contact was the most common type of contact (n=156, 81.3%). The secondary attack rate was the highest for close contact household family at 21.4%. After excluding asymptomatic contacts, the rate of secondary attack was the highest for close contact travel. The secondary attack rate for all contact types was (family, travel, and meal) 19.4% and 31.8% after excluding asymptomatic contacts. The secondary attack rate was 5.0% for presymptomatic contacts and 0.0% for asymptomatic and symptomatic contacts.

In a comprehensive epidemiologic investigation conducted to identify the routes of transmission, Spa facility A (for female) was found to be the only place where Patients #1, #2, and #3 were present at the same time. Fifty-eight people including the three confirmed cases appear to have visited Spa facility A (for female) around the same hours (Supplementary Material 1). Fifty-one visitors excluding the three confirmed cases and four untested cases all tested negative for COVID-19. Of the 51 visitors who tested negative, three were symptomatic, six were from another region including the special disaster zones (Daegu and Gyeongsangbuk-do Provinces), one visitor was associated with an religion-related outbreak, and one was suspected of having been in contact with Patients #1, #2, and #3 (Ⓧ/Ⓧ’) based on the statements from Patients #1, #2, and #3 regarding their stay at Spa facility A (for female) (Supplementary Material 2). The secondary attack rate among these visitors excluding the untested cases was 5.9%.

## DISCUSSION

**Figure 3.**
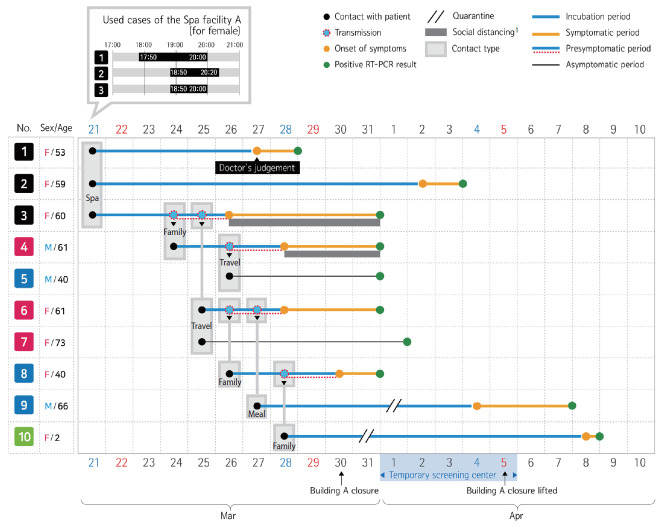
Timeline of events in the epidemiologically linked patients with coronavirus disease 2019 (COVID-19) in 2020. RT-PCR, reverse transcription polymerase chain reaction; F, female; M, male. ^1^Individuals did not leave home owing to symptoms such as fever and respiratory symptoms following the national guideline on Social distancing.

In this study, we examined how COVID-19 spread after an outbreak that started with 10 interconnected individuals at Spa facility A (for female) (Figure 3). Severe Acute Respiratory Syndrome (SARS) coronavirus and influenza viruses quickly lose survivability at high temperature and relative humidity [5,6] and are thus believed to lack transmissibility at high temperature and humidity. Therefore, one may think that these viruses have a low survivability in a spa where the temperature and humidity are high. However, we could not find any evidence that high temperature and humidity reduced the transmissibility of COVID-19 in our outbreak investigation. In our investigation, the secondary attack rate was 5.9% among the visitors of Spa facility A (for female) and 3.6% among all contacts. Thus, we cannot assume that the transmissibility of severe acute respiratory syndrome coronavirus 2 (SARS-CoV-2) is reduced in a spa where the temperature and humidity are high. Furthermore, since it was not possible to accurately determine how many people were in Spa facility A (for female) at the same time because of the limitations of an epidemiologic investigation, the number was estimated at 58 through a comprehensive epidemiologic investigation, and there is a possibility that the actual secondary attack rate is much higher than 5.9%. No additional confirmed cases of COVID-19 have been reported from the spa facility since the facility reopened on April 18, 2020.

COVID-19 is spreading throughout Southeast Asia where the climates are hot and humid [7]. There have also been occurrences of COVID-19 in spa facilities in other countries, suggesting the possibility of the environmental transmission of COVID-19 [7]. There are two possible explanations for how the outbreak occurred at Spa facility A. First, although there are claims that the transmissibility of COVID-19 decreases in hot and humid environments [8,9], the transmissibility of COVID-19 may not be significantly affected by temperature and humidity since the secondary attack rate among the visitors of the spa facility where the temperature and humidity were high was higher than that among all contacts. Second, since one cannot constantly maintain a 2 m distance from others and wear a mask inside a spa, it is more difficult to protect oneself from the direct transmission of COVID-19 through droplets. Based on the confirmed cases’ statements about their stay at Spa facility A (for female), there is a possibility of COVID-19 transmission through aerosols inside a whirlpool (similar to the transmission of Legionella through aerosols) [10,11], and through environments contaminated by droplets (e.g., doorknobs, etc.) [7]. Further research is needed on COVID-19 transmission through aerosols and contaminated environments.

We proposed three hypotheses to identify the patient zero of the outbreak. The first hypothesis was that Patient #3, who had the earliest symptom onset among Patients #1, #2, and #3, is the patient zero and transmitted the virus to other visitors. A Taiwanese study reported that COVID-19 can spread starting 1-4 days before symptom onset [13]. Based on this, Patient #3 was deemed unlikely to be the patient zero since the earliest she could start transmitting the virus would be March 22, 2020 (date of symptom onset: March 26, 2020). The second hypothesis was that Patients #1, #2, and #3 were infected via different routes. A comprehensive epidemiologic investigation was conducted in the places visited by Patients #1 and #3 (excluding Patient #2 who was confirmed with COVID-19 during the outbreak investigation), excluding Spa facility A, during the seven days before symptom onset (the mean incubation period of COVID-19 is 4.8 days according to a Chinese study [3]). An epidemiologic investigation and diagnostic test for COVID-19 were performed for suggested patient zeroes, but they all tested negative and showed no unusual epidemiological characteristics; thus, they were deemed unlikely to be the patient zero of the outbreak. The third hypothesis was that Patients #1, #2, and #3 were infected by an unknown patient zero at Spa facility A (for female), in which they stayed at the same time. Thus, an investigation was conducted to identify the patient zero among the visitors of Spa facility A (for female). The positive rate of RT-PCR detection of SARS-CoV-2 is the highest within one week after symptom onset and gradually decreases thereafter until negative conversion after three weeks. [12] Negative conversion is reported to occur earlier for upper respiratory tract samples than lower respiratory tract (sputum) samples [12]. Therefore, RT-PCR results at 2-3 weeks after the date of exposure to COVID-19 at the spa facility may not be meaningful. Four suggested patient zeroes—two residents of the special disaster zones (Daegu, North Gyeongsang Province), one person associated with a religion-related outbreak, and one suggested contact based on the statements of the confirmed cases (Ⓧ/Ⓧ’)—were thus selected based on their epidemiological links even though their RT-PCR results were negative. However, additional investigations were not conducted due to privacy issues.

Close contact with a confirmed case is known to be the greatest risk factor of COVID-19 transmission [10]. In our investigation, the secondary attack rate among close contacts (family, travel, meal) was 19.4%, and the secondary attack rate among all confirmed cases was 3.6%. The secondary attack rate among close contacts (family, travel) was high, consistent with a Chinese study [3]. Patient #3 transmitted the virus to a large number of people unlike Patients #1 and #2. This may be because Patient #3 had a higher ratio of close contacts (family, travel, meal) than Patients #1 and #2 (Patient #1: 2/37, Patient #2: 1/37, Patient #3: 7/20, Patient #6: 7/18) [3], although Patients #1 and #2 had a higher total number of contacts (n=37) than Patient #3 (n=20). In the present outbreak case, most contact occurred while confirmed cases were in the presymptomatic period, and no transmission occurred through initial contact during the symptomatic period. This finding was different from a previous finding in which COVID-19 was found to have high transmissibility starting from five days before the symptom onset [13]. As shown in Table 2, the number of presymptomatic contacts is 140 (close contacts: 34), and the number of symptomatic contacts is 52 (close contacts: 2), indicating a significant difference in the number of presymptomatic and symptomatic contacts. The mean time until isolation after the symptom onset among confirmed cases excluding asymptomatic patients was 2.1 days (1.7 days if asymptomatic patients are included) and was shorter than the time reported in a previous study of 3.43 days [14]. Based on these results, we believed that further transmission during the early symptomatic period could be prevented through rapid contact tracing and isolation by health authorities, prompt testing after symptom onset by a clinician, and social distancing [13]. In our investigation, no cases of asymptomatic transmission were found, and the secondary attack rate significantly increased when asymptomatic contacts were excluded (Table 2). Although it may be difficult to identify asymptomatic patients since COVID-19 can cause mild symptoms, research on asymptomatic transmission and its effect on an outbreak (e.g., transmissibility) is necessary. Lastly, while the secondary attack rate increases as the age of the contacts increase, it is also high among children and adolescents [3]. This suggests that children may also be susceptible to COVID-19 infection.

Our outbreak investigation has some limitations. First, the statistical significance of the differences in the secondary attack rate cannot be determined because of the small number of confirmed cases. Second, owing to the limitations of diagnostic test for COVID-19 (possibility of negative conversion of a sample over time and false negatives) and conventional contact tracing, it is possible that not all confirmed cases and contacts were traced. Third, the route of transmission and epidemiological links among the confirmed cases could not be clearly identified because the diagnostic test and investigation on the suspected patient zeroes of the outbreak at Spa facility A (for female) were delayed due to contact tracing, sample collection and visitor information gathering. In addition, it was not possible to identify the actual patient zero due to personal information protection issues. Fourth, there may be errors in the data regarding individuals’ exposure history to COVID-19 due to recall bias, and it is impossible to accurately determined when they were exposed to a family member confirmed with COVID-19 before or after the symptom onset.

In our study, we could see that COVID-19 can be transmitted presymptomatically unlike other known coronaviruses [15], and presymptomatic transmission is an important factor in the spread of SARS-CoV-2 as has also been demonstrated in Chinese and Singaporean modeling research [16]. To control an outbreak characterized by presymptomatic transmission and subclinical infection, comprehensive contact tracing and social distancing are crucial [14,16]. Therefore, to control COVID-19 outbreak, not only health institutions’ efforts, such as contact tracing, testing and isolation, but also general public’s efforts, such as personal hygiene practices and social distancing, are essential. Our outbreak investigation also showed that COVID-19 is likely transmitted through close contact such as contact with a family member and contact during travel rather than through casual contact. Therefore, measures against a pandemic such as prioritization of an epidemiologic investigation and contacts to be traced (e.g. prioritizing close contacts [family, travel, etc.] over casual contacts, contacts immediately before the symptom onset and presymptomatic contacts) may be necessary .

## Figures and Tables

**Table 1.** Clinical and epidemiological characteristics of epidemiologically-linked patients with coronavirus disease 2019 (COVID-19)

Characteristics	Patient ID									
	#1	#2	#3	#4	#5	#6	#7	#8	#9	#10
Sex	F	F	F	M	M	F	F	F	M	F
Age (yr)	53	59	60	61	40	61	73	40	66	2
Smoking status	-	-	-	-	-	-	-	-	-	-
Underlying disease	+ (diabetes)	-	-	+ (gout)	-	+ (thyroid cancer)	-	-	+ (hypertension)	-
Incubation period (d)^1^	6	12	5	4	Asymptomatic	3	Asymptomatic	4	8	11
No. of contacts (close contacts)	37 (2)	37 (1)	20 (7)	5 (1)	19 (14)	18 (7)	33 (0)	22 (3)	1 (1)	0 (0)
Time from symptom onset to isolation (d)	1	1	5	3	0	3	0	1	0	0
Symptom type										
Fever	+ (took an anti-pyretic)	-	+	+	-	- (took an anti-pyretic)	-	- (took an anti-pyretic)	-	+ (took an anti-pyretic)
Cough	+	+	-	-+	-	+	-	-	+	-
Sputum	-	-	-	-	-	-	-	-	+	-
Chills	+	-	-	+	-	+	-	-	-	-
Muscle pain	+	-	-	+	-	-	-	-	-	-
Sore throat	+	-	-	-	-	+	-	+	+	-
Dyspnea	-	-	-	-	-	-	-	-	-	-
Others	Lethargy	Lethargy	Lethargy	Rhinorrhea	-	-	-	-	-	Rhinorrhea

F, female; M, male; +, have symptom and history; -, have not symptom and history.

1 For “close contacts: household family,” transmission is assumed to have occurred through a contact two days before the symptom onset.

**Table 2.** The number of confirmed cases and contacts and secondary attack rates according to the contact type, symptom status, and age and sex distribution

Variables	No. of confirmed cases	No. of contacts	Secondary attack rate (%)^1^
Total contact	7	192	3.6
Contact type			
Close contact	7	36	19.4
Family	3	14	21.4
Travel	3	16	18.8
Meal	1	6	16.7
Casual contact	0	156	0.0
Contact type (excluding asymptomatic contacts)			
Close contact	7	22	31.8
Family	3	12	25.0
Travel	3	5	60.0
Meal	1	5	20.0
Casual contact	0	118	0.0
Symptom status			
Asymptomatic contact	0	52	0.0
Asymptomatic close contact	0	14	0.0
Presymptomatic contact	7	88	8.0
Presymptomatic close contact	7	20	35.0
Symptomatic contact	0	52	0.0
Symptomatic close contact	0	2	0.0
Age and sex distribution			
Age (yr)			
0-19	1	13	7.7
20-39	0	63	0.0
40-59	2	83	2.4
≥60	4	33	12.1
Sex			
Male	3	63	4.8
Female	4	129	3.1

1 Secondary attack rate (%)=number of confirmed cases/number of contacts×100.

## SUPPLEMENTARY MATERIALS

Supplementary Material 2.

Korean version is available at http://www.e-epih.org/.
