# Supplementary material for: Outbreak investigation: transmission of COVID-19 started from a spa facility in a local community in Korea
Source: Epidemiol Health. 2020 Jul 29;42:e2020056. doi: 10.4178/epih.e2020056 (PMC7871164; doi:10.4178/epih.e2020056)
Supplement: Supplementary Material 1. [file epih-42-e2020056-suppl1.pdf]

Volume: 42, Article ID: e2020056

https://doi.org/10.4178/epih.e2020056

Supplementary Material 1. Analysis table and result for 58 visitors to women's Spa facilities A from 17:00 to 20:00 based on CCTVs at the entrance

17:05 1 ● (-)
17:10 1 ○ (-) symptomatic
17:10 1 □ (-) Special disaster area
17:10 1 ● (-)
17:10 1 ■ (-) Special disaster area
17:30 2 □ (-/-) Other area
17:38 1 ● (-)
17:39 1 ● (-)
17:41 1 □ (-) Other area
17:42 1 ● (-)
17:42 1 ● (-)
17:42 1 ● (-)
17:42 2 ◇ (+/-) ①/①' -> 19:58 exit
(① 'is a family of ①)
17:43 2 ■ (-/-)
18:04 1 ● (-)
18:04 1 ○ (-)
18:09 1 ◆ (-)
18:09 1 ● (-)
18:10 1 ● (-)
18:10 1 ● (-)
18:10 2 ■ (-/-)
18:18 1 ● (-)
18:18 1 ● (-)
18:19 1 ■ (-) Ⓧ/Ⓧ'(suspected contact with ①,②,③)
18:20 1 ● (-)
18:20 1 ● (-)
18:25 1 ● (-) Related outbreak(Religious cluster) group
18:26 1 □ (-)
18:28 1 ● (-)
18:29 1 ● (-)
18:36 1 ● (-)
18:37 1 ● (-)
18:37 1 ● (-)
18:40 1 ● (-)
18:41 1 ◇ (?)
18:41 1 □ (-)
18:43 1 ● (-)
18:50 1 ● (-) symptomatic
18:52 1 ● (+) ③ -> 19:56 exit
18:54 1 ● (+) ② -> 20:20 exit
18:54 1 □ (-)
19:13 1 ● (-) symptomatic
19:15 1 ● (-)
19:16 1 ● (-)
19:20 1 ● (-)
19:34 1 ● (-)
19:39 1 ○ (-)
19;39 1 ● (-)
19:41 1 □ (?)
19:44 2 □ (?/?)
19:49 1 ◆ (-)
19:52 1 ◆ (-)
19:59 1 ■ (-) Other area

Analysis result

***Entrance detail**

Membership(●):34
Checking face(acquaintance)(○):3
Credit card(□):9
Cash(■):6
Coupon(◇):3
Staff(◆):3

***Test result**

positive(+):3
unidentified(?):4
negative(-):51

Volume: 42, Article ID: e2020056

https://doi.org/10.4178/epih.e2020056

***Epidemiological investigation**
(About 51 negatives)
Symptomatic:3
Residents of Other area:6(Special disaster area:2)
Related outbreak(religious cluster) group:1
Ⓧ/Ⓧ'(suspected contact with ①,②,③):1
