# Supplementary material for: Outbreak investigation: transmission of COVID-19 started from a spa facility in a local community in Korea
Source: Epidemiol Health. 2020 Jul 29;42:e2020056. doi: 10.4178/epih.e2020056 (PMC7871164; doi:10.4178/epih.e2020056)
Supplement: Supplementary Material 2. [file epih-42-e2020056-suppl2.pdf]

[20-30 persons in the spa facility based on statement; entrance/exit time: based on CCTV in counter area]

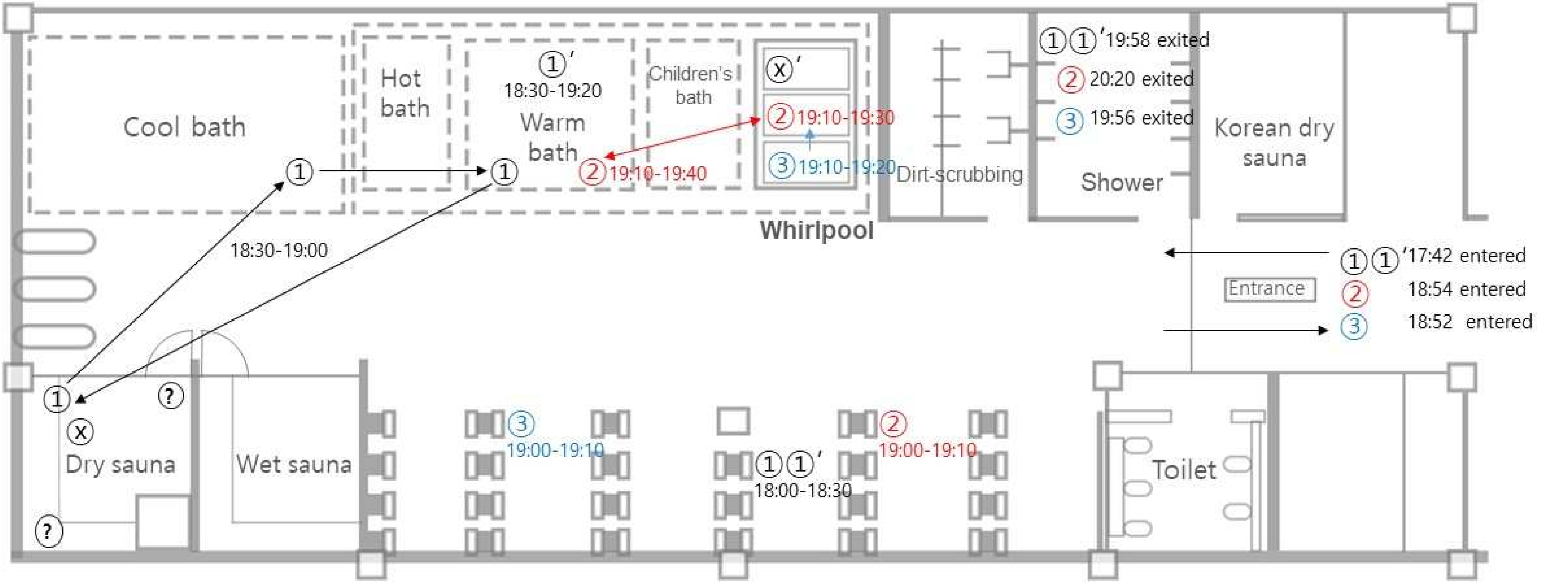

[other things to check]

①①′ (patient 1/family): dryer(o),water purifier(x), store(o

②(patient 2): dryer(o), water purifier(o), store(x)

③(patient 3): dryer(x), water purifier(o), store(x)
