# Supplementary material for: Outbreak investigation: transmission of COVID-19 started from a spa facility in a local community in Korea
Source: Epidemiol Health. 2020 Jul 29;42:e2020056. doi: 10.4178/epih.e2020056 (PMC7871164; doi:10.4178/epih.e2020056)
Supplement: Supplementary file 3 [file epih-42-e2020056-suppl3.docx]

**Outbreak investigation: transmission of COVID-19 started from a spa facility in a local community in Korea**

**Taewon Han**

**Local Epidemic intelligence Officer and Public Health Doctor, Gyeongsangnam-do, Korea**

**ABSTRACT**

**BACKGROUND**

2019년 12월 중국, 우한시에서 시작된 새로운 코로나바이러스가 전세계적으로 대유행을 형성하였다. 대한민국에서는 2020년 1월20일 첫 환자가 확진 된 이후로 2020년 4월11일까지 10480명의 사례가 발생하였다. 우리는 대한민국의 지역A의 목욕탕A에서 시작된 집단발병과 지역사회로의 전파과정을 통해서 증상발현 이전 전파의 중요성 등에 대해 알아보았다.

**METHODS**

2020년 3월28일 확진된 환자를 시작하여 2020년 4월8일까지 대한민국의 지역A의 목욕탕A와 관련된 10명(남성:3명, 여성7명, 2세~73세)의 코로나19 확진자들의 역학적 특성을 분석하였다. 각 확진자들의 접촉자들과 감염원파악을 위해 확진자들과 동일한 시간대에 목욕탕A(여)를 이용한 방문자들에 대해서 역학조사 및 확진검사를 진행하고, 이에 기반하여 2차 감염률을 추정하였다.

**RESULTS**

확진자의 성별 분포는 남성 3명(30%) 여성 7명(70%)이며 평균 연령은 53.5세(2세~73세)이고 무증상자는 2명(20%)이다. 잠복기는 3일에서 12일사이에 분포하고 있다. 목욕탕에서 발생한 확진자는 3명이며, 7명은 목욕탕 확진자의 2차, 3차, 4차 감염자(2^nd^,3^rd^,4^th^ generation)이다. 2차, 3차, 4차 감염자(2^nd^,3^rd^,4^th^ generation) 7명 모두는 전증상 접촉을 통한 전파(Presymptomatic transmission)였다. 총 192명의 접촉자가 확인되었고, 이차발병률은 3.6%이다. 접촉자는 40-59세사이가 83명(43.2%)으로 가장 많았고 이차발병률은 60세 이상에서 12.1%로 가장 높았다. 노출력에 따른 접촉자수는 일반접촉이 156명(81.3%)으로 가장 많았다. 목욕탕A(여)를 이용한 방문자들은 확진자 3명을 포함하여 총 58명이며, 이차발병률은 5.9%이다.

**CONCLUSIONS**

본 연구를 통해 온도와 습도가 높은 장소에서도 집단감염이 발생하는 예시를 확인할 수 있었다.목욕탕A(여)에서 시작된 본 집단발병의 경우 2-4차 감염의 전수는 증상발현 이전의 밀접 접촉을 통해 일어났다. 이러한 전증상 전파 및 Subclinical infection 등이 특징인 유행을 조절하기 위해서는 개인위생 및 사회적 거리 두기, 각 지자체 및 보건당국의 빠른 접촉자 파악과 격리 그리고 의사의 적절한 판단을 통한 빠른 검사 등이 필요하다.

**KEWORD**

: COVID-19, Transmission, Disease outbreak, Contact tracing, Korea

**Introduction**

2019년 12월 중국 우한에서 시작된 이후 코로나19는 전세계적으로 퍼져나가 2020년 3월 11일 세계보건기구(World health Organization)는 대유행(Pandemic)을 선언하였다. 코로나19는 발열, 기침, 근육통 등을 증상으로 하며 전세계적으로 많은 확진자 및 사망자들이 발생하고 있다.[1] 대한민국에서는 2020년 1월20일 중국 우한시에서 입국한 사람이 처음 확진 된 이후 2020년 4월 11일 기준 10480명의 확진자가 확인되었고, 211명이 사망하였다. 2월말~4월초까지 확진된 대부분의 사례는 정부에서 특별재난지역으로 선포한 대구, 경북에서 발생하였으며, 현재는 전국에서 산발적으로 발생하는 경향을 보이고 있다.[2]

전 세계적으로 시행된 많은 연구를 바탕으로 코로나19의 자연사 및 전파경로의 중요한 측면이 밝혀지고 있으나 여전히 의문점들이 남아있다. 그 중 하나는 무증상 또는 전증상 전파의 가능성과 그 영향력이다.[3] 코로나19는 이전의 코로나바이러스와 달리 증상 초기 높은 바이러스 부하를 가진다. 무증상 환자에서 검출된 바이러스 부하 역시도 유증상 환자와 유사하며 이를 통해 무증상 또는 감염과정 초기에 전염이 일어날 수 있을 것을 추측할 수 있다.[4] 증상 발현 이전에 감염력이 있어 전파가 시작된다면, 증상이 발현한 환자를 빠르게 검사하고 격리(Isolation)하는 것뿐 만 아니라 증상 발현 이전 또는 증상이 경미한 확진자를 적극적으로 찾아내고 확진자의 접촉자를 선제적으로 자가격리(Quarantine)하는 것이 필요할 것이다. 그러나 이는 현실적으로 많은 어려움이 따를 것이다.[3]

본 연구에서는 대한민국의 지역A에서 발생한 집단발병 사례를 바탕으로 코로나19의 증상 발현 전 전파 양상과 접촉자 추적(Contact tracing)을 통해 코로나19의 전파를 어느정도 조절할 수 있었는지를 알아보고자 한다. 또한 추적을 통해 설정한 접촉자들을 바탕으로 지역사회 집단발병 사례에서의 코로나19 유행의 특징과 이차발병률 등의 정보를 알아보았다.

**Methods**

**Background and Setting)** 2020년 3월28일부터 3월31일까지 다수의 코로나19 확진자가 지역A에서 발생하였고 지역A에 위치한 빌딩A 및 다중이용시설(목욕탕A)과 연관이 있음을 확인하였다. 지역A는 인구 약 35만명의 도시이며 주변 도시들의 행정, 교통 및 생활권 거점도시의 역할을 하고 있다. 이번 목욕탕A관련 집단발병 이전 대구 신천지교회 관련 확진자 2명이 약 40일 전 발생한 것 외에 확진자가 발생한 적이 없는 지역이다. 빌딩A은 지하3층에서 지상19층의 규모로 상업시설(지하1층~지상1층)과 지식산업센터(2층~19층)가 입주하고 있다. 상주 인원은 약 2500명이며 1일 출입차량은 약 2300대이다. 상업 시설의 방문객을 포함한 유동인구는 하루에 약 350명이다. 목욕탕A은 빌딩A의 지하1층에 위치하고 있고, 남녀 목욕탕은 분리되어 공용으로 사용하는 공간은 없다. 일일 방문자는 약 250명이며 피트니스센터와 함께 정기적으로 이용하는 사람들이 많고 주말엔 타 지역에서 방문하는 사람들도 많다. 따라서 지역 보건소와 시도 역학조사반은 집단발병 및 감염원(Patient zero) 파악과 확진자의 접촉자 추적(Contact tracing)을 위해 합동역학조사반을 구성하여 역학조사를 진행하였다.

**Epidemiologic investigation and Response Measures)** 첫번째 확진자(Index case)인 환자#1은 3월27일부터 발생한 발열, 근육통 및 무기력증 등의 증상으로 A의원을 방문하였다. 코로나19의 검사가 필요하다는 의사의 소견에 따라 보건소 선별진료소를 내원하여 검사를 받아 3월28일 확진 되었다. 특별재난지역(대구,경북) 및 해외방문 또는 확진자와의 접촉력 등의 역학적 연관성이 없어 감염원을 알 수 없는 환자였다. 이후 3월31일, 5일 전부터 발생한 발열 및 무기력증 증상으로 병원 선별진료소에서 검사 받은 환자#3과 환자#3의 배우자로 발열 및 근육통이 3일 전부터 발생한 환자#4가 동시에 확진 되었다. 환자#3, #4 모두 앞의 환자#1과 같이 역학적 연관성이 없어 감염원을 알 수 없는 환자였다. 이후 접촉자 조사(Contact tracing)를 통해 환자#5~#10이 확진 되었다. 감염원(Patient zero)을 찾는 심층역학조사결과 환자#1,#3,#4가 빌딩A의 다중이용시설(목욕탕A)을 주기적으로 방문함이 확인되었고 환자#4,#5의 근무지가 빌딩A에 있는 것으로 파악되었다. 이에 빌딩A와 관련한 집단발병으로 생각되어 빌딩A 방문자에 대한 검사 및 역학조사를 진행하였고 4월3일 환자#2가 확진 되었다. 빌딩A은 집단발병이 보고된 3월31일부터 폐쇄조치 하였고 빌딩A 앞에 임시선별진료소를 4월1일부터 4월5일까지 설치하여 빌딩A 입주자 및 방문자들이 선별진료 및 검사를 받을 수 있게 하였다. 지역A 외 방문자는 인근 지역으로 재난문자를 발송하여 거주지 보건소에서 검사를 받도록 하였다. (증상발생일이 가장 빠른)환자#3의 증상발생일 기준 14일 전인 3월12일부터 빌딩A 폐쇄일인 3월31일까지 빌딩A를 방문한 사람 전수를 대상으로 조사를 시행하였고 검사 대상자는 1. 3월12일~3월31일 직원, 방문자, 기숙사 거주자 중 유증상자 2. 3월12일부터 3월24일까지 목욕탕A 방문자 3. 3월27일~3월31일 확진자 들의 사무실과 같은 층에서 근무한 사람 4. 그 외 앞에서 언급한 접촉자 전수에 대해 검사를 시행하였다. 검사대상자들에 대한 코로나19 확진검사는 Real time reverse transcription PCR assays를 통해 진단하였고 이를 위해 상기도(비인두, 구인두) 및 하기도 (객담) 검체를 채취하였으며 객담이 없는 경우 하기도 검체는 채취하지 않았다. 확진자는 감염병전담 병원 이송 및 역학조사를 진행하였고, 코로나19 검사에서 음성판정을 받은 자는 1.은 증상 소실 시 2.은 검사결과가 나올 때까지 3,4.은 마지막 노출일로부터 14일간 자가격리(Quarantine)를 시행하였다. 감염경로 파악을 위해 환자#1,#2,#3의 위성항법시스템(GPS, Global positioning system), 카드사용내역서, 심층면담 등을 통해 심층역학조사를 진행하였다. 환자#1,#2,#3과 동일한 시간대에 목욕탕A(여)를 방문하였을 것으로 판단되는 사람들을 폐쇄 회로 텔레비전(CCTV, Closed circuit television), 입장기록 등을 통해 신원을 파악하여 방문자로 설정하였으며 이들에 대해 전화역학조사와 확진검사를 진행하였다.

**Case definition)** 확진자는 코로나19 검사에서 양성판정을 받은 사람으로 정의하였다. 증상 후 접촉(유증상접촉, Symptomatic contact)은 증상이 있었던 확진자와의 접촉으로 정의하였으며 증상 전 접촉(전증상접촉, Presymptomatic contact)은 접촉당시에는 증상이 없었으나 이후 증상이 발생한 확진자와의 접촉으로 정의하였다. 무증상환자(Asymptomatic patient)는 접촉시점부터 검사시행까지 증상이 없고 코로나19 검사에서 양성판정을 받은 사람으로 정의하였으며 무증상환자와 접촉한 경우 무증상접촉(Asymptomatic contact)으로 정의하였다. 각 접촉에 의한 전파를 유증상전파(Symptomatic transmission), 전증상전파(Presymptomatic transmission), 무증상전파(Asymptomatic transmission)로 정의하였다. 접촉 및 각 접촉에 의한 전파는 최초접촉일을 기준으로 분류하였다. 밀접접촉:가족(Household family)의 경우는 동거가족으로 정의하였고 밀접접촉:여행(Travel)의 경우 목적에 관계없이 확진자와 지역A 이외의 지역으로 이동하여 3시간 이상 밀접하게 머무른 경우, 밀접접촉:식사(Meal)는 식사와 더불어 30분이상 밀접하게 접촉한 경우로 정의하였다. 그 외 일반접촉(Casual contact)은 마스크를 착용하지 않고 확진자와 동일한 공간, 수분 이상 머무른 경우(또는 머물러 역학조사관이 접촉자로 설정한 경우)로 정의하였다. 분류가 겹치는 경우 가족, 여행, 식사, 일반접촉 순으로 분류하였다. 접촉자는 증상에 관계없이 접촉력이 확인된 경우로 정의하여 코로나19 검사를 진행하였고, 검사결과 음성인 경우 확진자와 마지막 접촉일로부터 14일간 자가격리(Quarantine)를 실시하였다. 자가격리(Quarantine) 중 증상이 생기거나 변화하는 경우 추가검사를 진행하였고 가족 및 의료기관 관련자 등의 경우 자가격리 해제 전 검사를 시행하였다.

**Data Collection and Analysis)** 우리는 질병관리본부에서 제공하고 지역 보건소에서 수정한 표준화된 역학조사양식을 사용하였고 위성항법시스템(GPS, Global positioning system), 카드사용내역서, 폐쇄 회로 텔레비전(CCTV, Closed circuit television) 등 으로 객관적인 정보를 얻었다. 전화인터뷰를 통해 역학적 연관성과 증상 유무 등에 대한 정보를 얻었고 이를 바탕으로 기술 분석을 수행하였다.

**Result**

2020년 3월28일부터 4월3일까지 환자#1부터 환자#8까지 총 8명의 확진자가 발생하였고 이후 자가격리(Quarantine)중이었던 환자#9,#10는 증상발현으로 검사하여 추가로 확진 되어 총 10명의 환자가 확인되었다. 이번 집단발병 사례의 역학곡선은 Figure 1과 같다. 3월12일 - 3월31일의 기간 동안 빌딩A를 방문한 것으로 확인된 2843명 중 2245명이 코로나19 검사를 받았으며 그 중 4월3일 1명(환자#2)이 확진 되었다. 확진자들의 역학조사를 통해 설정된 접촉자는 총 192명이었고 그 중 7명이 확진 되었다. 목욕탕 관련 집단발병에서 발견된 10명 환자의 임상적인 특징은 Table1에 나타내고 있다. 성별은 남성 3명(30%), 여성 7명(70%)이고 평균 연령은 53.5세(2세~73세)이고 무증상자는 2명(20%)이다. 잠복기는 3일에서 12일사이에 분포하고 있다. 환자의 접촉자 수의 분포는 0-37명이며, 이 중 밀접접촉자로 분류된 사람은 0-14명이었다. 증상은 발열, 기침, 인후통, 오한 등의 순서로 빈번하였다. 이 중 3명의 확진자(환자#1,#2,#3)는 역학조사를 통해 빌딩A의 목욕탕A(여)을 동일시간대에 방문함을 확인하였다.

환자 10명의 추정 감염경로는 Figure 2와 같다. 환자#1,#2,#3은 3월21일 모두 목욕탕A(여)에서 알수 없는 감염원(Patient zero)에게 공동 노출되어 감염된 것으로 추정된다. 환자#3이 환자#4(가족), 환자#6(여행), 환자#7(여행)에게 전파시키고 환자#4가 환자#5(여행)에게 전파시켰다. 환자#6은 환자#8(가족), 환자#9(식사)에게 전파시켰고 환자#8는 환자#10(가족)에게 전파시킨 것으로 추정된다. 환자#1과#2의 경우 2차 전파를 일으키지는 않았다.

Table 2는 192명 접촉자들의 접촉형태와 특징 별 이차발병률을 나타내고 있다. 이 집단에서의 평균 이차 발병률은 3.6%이다. 접촉자는 여성이 많았으며 (남성이 32.8%, 여성 67.2%), 성별에 따른 이차발병률은 남성 4.8% 여성 3.1%이다. 접촉자의 연령은 40-59세사이가 83명(43.2%)으로 가장 많았으나 이차발병률은 60세 이상에서 12.1%로 가장 높았다. 접촉형태에 따른 접촉자수는 일반접촉이 156명(81.3%)으로 가장 많았다. 접촉형태에 따른 이차발병률은 밀접접촉:가족인 경우가 21.4%로 가장 높았으며, 무증상 접촉에 의한 접촉자(Asymptomatic contact)를 제외하였을 경우는 밀접접촉:여행인 경우가 60.0%로 가장 높았다. 전체 밀접접촉(가족, 여행, 식사)의 이차발병률은 19.4%이며 무증상접촉에 의한 접촉자(Asymptomatic contact)를 제외하였을 경우는 31.8%이다. 증상 전 접촉의 이차 발병률은 5.0%이며 무증상 접촉과 증상 후 접촉의 이차 발병률은 0.0%이다.

감염경로 파악을 위한 심층역학조사 결과 3월1일부터 3월31일까지 환자#1,#2,#3의 동선 및 시간이 겹치는 곳은 해당 시간 목욕탕A(여) 이외에 없었다. 동일한 시간대에 목욕탕A(여)을 방문하였을 것으로 판단되는 방문자는 확진자 3명 포함 58명이다.(Appendix1) 확진자 3명과 미검사자 4명을 제외한 51명에 대한 코로나19 확진검사는 전부 음성이었으며 음성 51명 중 유증상자 3명, 특별재난지역 거주자(대구,경북) 2명 포함 타지역거주자 6명, 종교관련 집단발생연관자 1명, 환자#1,#2,#3 목욕탕A(여) 이용진술(Appendix2)에 따른 환자#1,#2,#3의 공통접촉 의심자(Ⓧ/Ⓧ’) 1명이었다. 이를 통해 계산한 목욕탕A(여) 내 미검사자를 제외한 이차 발병률은 5.9%이다.

**Discussion**

우리는 목욕탕A(여)에서 시작된 10명의 서로 연결된 코로나19 유행을 통해 코로나19의 전파가 어떤 식으로 일어나는지 분석하였다. 기존의 SARS 코로나 바이러스와 인플루엔자 바이러스의 생존력은 높은 온도와 높은 상대습도에서 빠르게 손실되며 [5, 6] 그렇기에 높은 온도와 습도에서는 전파력이 떨어진다고 알려져 있다. 이에 목욕탕과 같이 온도와 습도가 높은 곳에서는 바이러스의 생존력이 낮아 상대적으로 안전하다고 생각할 수 있다. 하지만 우리들의 유행조사를 바탕으로 높은 온도와 습도에 의해 코로나19의 전파력이 감소한다는 근거는 찾을 수 없었다. 우리 조사에서의 목욕탕A(여)에 대한 이차발병률은 5.9%이며 전체 접촉자에 대한 이차발병률은 3.6%로 온도와 습도가 높은 목욕탕이라고 하여 SARS-CoV-2의 전파가 줄어든다고 판단하기 어렵다. 또한 실제 역학조사의 한계로 인해 확진자들과 동시에 몇 명이 목욕탕A(여)를 이용하였는지 정확히 알기 어려워 목욕탕A(여)를 동시에 이용하였을 가능성이 높은 58명을 심층역학조사를 통해 광범위하게 정하였기 때문에 실제 이차 발병률은 5.9%보다 훨씬 높을 수도 있다. 이후 목욕탕A가 영업을 재개한 4월18일까지 해당시설에서 코로나19 추가 확진자는 발생하지 않았다.

코로나19의 경우 온도와 습도가 높은 동남아시아에서도 확산되고 있고[7] 다른 나라의 목욕시설에서 코로나19의 발생 사례가 있어 환경을 통한 전파의 가능성이 존재한다.[7] 이번 목욕탕에서 의 집단감염은 두가지로 해석할 수 있다. 첫째로 온도와 습도가 높아지는 계절에는 코로나19의 감염력이 감소할 수 있다는 주장이 있으나,[8, 9] 본 연구 결과 온도와 습도가 높은 목욕탕에서의 이차발병률이 전체 접촉자에 비해 높다는 것을 고려하였을 때 코로나19의 감염력은 온도와 습도에 영향을 많이 받지 않는다고 할 수 있다. 둘째로 목욕탕의 경우 2M 이상 거리유지가 어렵고 마스크를 착용할 수 없어 비말의 직접전파에서 개인을 보호하기 어렵다는 것이다. 확진자들의 목욕탕A(여) 이용진술(Appendix1)을 바탕으로 (레지오넬라균의 전파와 같이) 월풀을 통한 에어로졸의 형성에 따른 전파 [10, 11] 및 비말에 의해 오염된 환경(손잡이 등)의 접촉전파의 가능성도 생각해 볼 수 있다.[7] 이러한 에어로졸 및 오염된 환경에 의한 전파에 대해서는 추가적인 연구가 필요할 것이다.

우리는 감염원(Patient zero)을 찾기 위해 3가지의 가설을 설정하였다. 첫째로 환자#1,#2,#3들 중 증상발현일이 가능 빠른 환자#3이 감염원(Patient zero)이고 그를 통한 전파 가능성이다. 대만의 연구에 따르면[13], 증상발현 1~4일 이전부터 전파력이 있다. 따라서 환자#3의 전파가능일은 증상발현일 최소 3월22일(증상발현일:26일) 이후이기에 가능성이 낮다. 둘째로 환자#1,#2,#3이 각자 다른 경로로 감염되었을 가능성이다. 집단발병 조사를 통해 확진된 환자#2를 제외하고 환자#1,#3의 증상발현일 7일 이전(중국의 연구[3]에 따르면 평균잠복기는 4.8일) 방문지(목욕탕A를 제외한)에서의 심층역학조사(감염원 조사)를 진행하였다. 심층역학조사결과 설정된 감염원 의심자(Suspected patient zero)에 대한 역학조사 및 확진검사 진행하였으나 전부 음성판정을 받았고 역학적으로 특이사항은 없었기에 가능성이 낮다고 판단하였다. 그래서 셋째로 우리는 환자#1,#2,#3이 유일하게 동일한 시간대에 방문한 장소인 목욕탕A(여)에서 알 수 없는 공통 감염원(Patient zero)에 의해 감염되었을 것이라 추정하였다. 따라서 목욕탕A(여)내 감염원(Patient zero)을 파악하기위한 조사를 진행하였다. 코로나19의 확진검사인 RT-PCR의 경우 양성률이 증상발현 후 1주차내에 가장 높고 점차 감소하여 3주 이후 대부분 음전 되며, 상기도검체의 경우 하기도(객담)검체보다 더 빠르게 음전 되는 것으로 알려져 있다. [12] 이에 목욕탕 노출일로부터 2~3주가 지난시점의 검사결과는 의미가 적기에 검사결과 음성이지만, 역학조사를 통해 알게 된 역학적연관성 등을 바탕으로 특별재난지역(대구,경북) 거주자 2명, 종교관련 집단발생관련자 1명, 확진자들의 진술을 통한 목욕탕A(여) 내 공통접촉 의심자(Ⓧ/Ⓧ’) 1명 총 4명의 감염원 의심자(Suspected patient zero)를 설정하였다. 하지만 개인정보보호 등의 문제로 인해 추가적인 조사는 진행하지 못하였다.

코로나19는 확진자와 밀접하게 접촉하는 것이 전파의 가장 큰 위험인자라고 잘 알려져 있다.[10] 우리의 조사에서도 밀접접촉(가족, 여행, 식사)을 통한 이차발병률은 19.4%이고 전체 이차발병률 3.6%로 이전 중국의 연구와 마찬가지로 가족, 여행 등 밀접한 접촉에서 높은 이차발병률을 보이고 있다. [3] 또한 환자#1,#2와 다르게 환자#3으로부터 많은 전파가 일어났는데 이는 전체 접촉자수가 37명인 환자#1,#2 보다 전체접촉자수는 20명으로 적지만 환자#3의 경우 환자#1,#2 보다 밀접한 접촉자의 비율(가족, 여행, 식사)이 높기에 많은 전파가 발생 할 수 있었을 것이다. (환자#1: 2/37, 환자#2: 1/37, 환자#3: 7/20, 환자#6: 7/18)[3]. 또한 우리의 유행에서는 대부분의 접촉이 확진자들의 전증상기에 발생하였으며 유증상기의 최초접촉을 통한 전파는 발생하지 않았다. 연구를 통해 알려진 증상 발현 직후부터 5일 전까지의 코로나19의 전파력이 높은 것과는 조금 다른 양상을 보이고 있다.[13] Table2를 보면 증상 발현 전 접촉자의 수가 140(밀접접촉:34)명이고 증상 발현 후 접촉자의 수가 52(밀접접촉:2)명인 것을 통해 증상 전 후 접촉자 수의 상당한 차이가 있다는 것을 알 수 있다. 그리고 확진자의 증상발현 이후 격리(Isolation)까지의 평균기간은 무증상자를 제외하고 2.1일(무증상자 포함 1.7일)로 이전 연구에서 제시한 3.43일보다 짧은 것을 알 수 있다.[14] 이를 통해 보건당국의 빠른 접촉자 추적(Contact tracing)과 격리(Isolation), 임상의사의 적절한 판단을 통한 증상발현 후 빠른 검사 그리고 사회적 거리두기 등을 통해 유증상기 초기의 추가적인 전파를 막을 수 있었다고 생각하였다.[13] 또한 우리 조사에서는 무증상접촉에 의한 전파(Asymptomatic transmission)는 확인되지 않았으며 무증상접촉에 의한 접촉자(Asymptomatic contact)을 제외한 경우 이차발병률 역시 크게 상승하는 것을 알 수 있다.(Table2) 경미한 증상 등도 특징으로 하는 코로나19에서 실제 무증상환자(Asymptomatic patient)를 가려내기는 어려울 것이지만 무증상환자의 전파(Asymptomatic transmission)와 유행에 미치는 영향(전파력 등)에 대해서 연구가 필요할 것이다. 마지막으로 접촉자들의 나이가 증가할수록 이차발병률이 증가하는 양상을 보이지만 소아 및 청소년의 경우도 이차발병률이 높음을 알 수 있다.[3] 이는 소아환자 역시도 코로나19의 감염에 취약할 수 있다는 근거를 제시한다.

우리의 유행조사에는 몇가지 한계가 있다. 1. 확진자 수가 적어 통계학적으로 각 이차발병률 차이등 의 유의성을 입증하기 어렵다는 점 2. 검사(시간에 따른 검체 음전률 및 위음성 가능성 등)와 전통적인 접촉자 추적(Conventional Contact tracing)의 한계로 인해 확진자 및 접촉자를 놓쳤을 가능성 3. 접촉자 파악 및 검체채취, 방문자들에 대한 정보확인 등의 이유로 목욕탕A(여)내 감염원 의심자(Suspected patient zero)에 대한 검사 및 조사가 지연되었고 개인정보보호 등으로 인해 실제 감염원(Patient zero)을 찾아내지 못하여 목욕탕A(여)에서의 전파 경로 및 확진자들 간의 확실한 감염의 연결고리를 알 수가 없었다는 점 4. 개인의 기억에 한계가 있어 노출력에 오류 가능성이 있고 가족 등의 접촉자의 경우 증상발현 전과 후 정확히 언제 노출이 이루워졌는지 알 수 없다는 점이 있다.

우리의 사례에서 코로나19는 이전의 다른 코로나바이러스와는 다르게 전증상 전파(Presymptomatic transmission)가 가능하며[15], 중국과 싱가포르의 모델링 연구와 마찬가지로 이러한 특성이 SARS-CoV-2의 확산에 중요한 요인임을 확인할 수 있었다.[16] 전증상 전파(Presymptomatic transmission) 및 Subclinical infection 등이 특징인 유행을 조절하기 위해서는 완벽한 접촉자 추적(Contact tracing)뿐만 아니라 사회적거리두기 역시도 필수적이다.[14, 16] 그러므로 접촉자 추적(Contact tracing)과 검사 및 격리(Isolation) 등의 보건기관의 노력과 더불어 개인위생수칙 및 사회적거리두기 등의 시민들의 노력이 반드시 필요할 것이다. 또한 우리의 유행조사 결과 일반적인 접촉에 의한 전파보다 가족, 여행 등의 밀접한 접촉을 통한 전파의 가능성이 높음을 알 수 있다. 따라서 대유행 발생시 역학조사 및 접촉자 추적(Contact tracing) 대상자의 우선순위 설정(증상발현 이전 및 직전, 일반적인 접촉보다 가족, 여행 등의 밀접한 접촉) 등의 대응 전략이 필요할 것이다.

**Acknowledgments**

우리나라 시군구 보건소의 감염병 대응 역량을 보여줄 수 있는 좋은 기회를 주신 최보율 교수님, 황혜경 소장님, 박보영 교수님께 감사드리며 앞으로도 시군구 보건소에서 감염병대응에 대한 자료 및 근거를 만들어 나갈 수 있도록 노력하겠습니다. 그리고 유행조사 및 대응에 도움을 주셨던 질병관리본부 이혜영, 현정희 선생님, 도청 감염병 관리지원단과 윤준우 선생님, 공중보건의사 문병선, 박경원, 위명철, 정영채, 제창호 선생님, 자료수집 및 정리 등에 도움을 주신 김진미, 최분자, 강상정, 하미경 선생님과 현장대응에 도움을 주신 감염병관리팀 및 시청, 보건소 직원분들 등 서로 다른 위치에 있지만 유기적인 협력을 통해 해결할 수 있다는 것을 보여주셔서 감사드립니다. 마지막으로 현재 코로나19 대응을 위해 최선을 다하고 계신 전국의 모든 역학조사관분들과 시군구 지역 보건소 및 공중보건의사 선생님들 그리고 환자분들의 치료에 힘쓰고 계신 의료진분들께 깊은 감사를 드리며, 이러한 노력을 통해 우리는 머지않아 해답을 찾을 수 있을 것이라 확신합니다

**References**

1. Li Q, Guan X, Wu P et al. Early Transmission Dynamics in Wuhan, China, of Novel Coronavirus-Infected Pneumonia. N Engl J Med 2020; 382: 1199-1207.

2. Korea Centers for Disease Control and Prevention. COVID-19 guidelines for local government 7-3th edition [cited 2020 Mar 15]. Available from: https://www.cdc.go.kr/board/board.es?mid= a20507020000&bid= 0019 (Korean).

3. Bi Q, Wu Y, Mei S et al. Epidemiology and transmission of COVID-19 in 391 cases and 1286 of their close contacts in Shenzhen, China: a retrospective cohort study. The Lancet Infectious Diseases.

4. Zou L, Ruan F, Huang M et al. SARS-CoV-2 Viral Load in Upper Respiratory Specimens of Infected Patients. N Engl J Med 2020; 382: 1177-1179.

5. Chan KH, Peiris JS, Lam SY et al. The Effects of Temperature and Relative Humidity on the Viability of the SARS Coronavirus. Adv Virol 2011; 2011: 734690.

6. Lowen AC, Mubareka S, Steel J, Palese P. Influenza virus transmission is dependent on relative humidity and temperature. PLoS Pathog 2007; 3: 1470-1476.

7. Luo C, Yao L, Zhang L et al. Possible Transmission of Severe Acute Respiratory Syndrome Coronavirus 2 (SARS-CoV-2) in a Public Bath Center in Huai'an, Jiangsu Province, China. JAMA Netw Open 2020; 3: e204583.

8. Sajadi MM, Habibzadeh P, Vintzileos A et al. Temperature and latitude analysis to predict potential spread and seasonality for COVID-19. 2020.

9. Wang J, Tang K, Feng K, Lv WJAaS. High temperature and high humidity reduce the transmission of COVID-19. 2020.

10. Bajema KL, Oster AM, McGovern OL et al. Persons Evaluated for 2019 Novel Coronavirus - United States, January 2020. MMWR Morb Mortal Wkly Rep 2020; 69: 166-170.

11. Brachman PS, Abrutyn E. Bacterial infections of humans: epidemiology and control. New York: Springer; 2009, p. 365.

12. Zheng S, Fan J, Yu F et al. Viral load dynamics and disease severity in patients infected with SARS-CoV-2 in Zhejiang province, China, January-March 2020: retrospective cohort study. 2020; 369: m1443.

13. Cheng HY, Jian SW, Liu DP et al. Contact Tracing Assessment of COVID-19 Transmission Dynamics in Taiwan and Risk at Different Exposure Periods Before and After Symptom Onset. JAMA Intern Med 2020.

14. Hellewell J, Abbott S, Gimma A et al. Feasibility of controlling COVID-19 outbreaks by isolation of cases and contacts. Lancet Glob Health 2020; 8: e488-e496.

15. Rothe C, Schunk M, Sothmann P et al. Transmission of 2019-nCoV Infection from an Asymptomatic Contact in Germany. N Engl J Med 2020; 382: 970-971. 16. Ganyani T, Kremer C, Chen D et al. Estimating the generation interval for coronavirus disease (COVID-19) based on symptom onset data, March 2020. Euro Surveill. 2020;25(17).
